# Neurohormetic phytochemicals in the pathogenesis of neurodegenerative diseases

**DOI:** 10.1186/s12979-022-00292-x

**Published:** 2022-08-11

**Authors:** Adeleh Sahebnasagh, Samira Eghbali, Fatemeh Saghafi, Antoni Sureda, Razieh Avan

**Affiliations:** 1 Clinical Research Center, Department of Internal Medicine, School of Medicine, North Khorasan University of Medical Sciences, Bojnurd, Iran; 2 Department of Pharmacognosy and Traditional Pharmacy, School of Pharmacy, Birjand University of Medical Sciences, Birjand, Iran; 3 Cellular and Molecular Research Center, Birjand University of Medical Sciences, Birjand, Iran; 4 Department of Clinical Pharmacy, Faculty of Pharmacy and Pharmaceutical Sciences Research Center, Shahid Sadoughi University of Medical Sciences, Yazd, Iran; 5 Research Group on Community Nutrition and Oxidative Stress, University of the Balearic Islands-IUNICS, and Health Research Institute of Balearic Islands (IdISBa), Palma de Mallorca, Spain; 6 CIBER Fisiopatología de la Obesidad y Nutrición (CIBEROBN), Instituto de Salud Carlos III (ISCIII), Madrid, Spain; 7 Department of Clinical Pharmacy, School of Pharmacy, Medical Toxicology and Drug Abuse Research Center, Birjand University of Medical Sciences, Birjand, Iran

**Keywords:** Hormesis, Neurodegenerative disorders, Neurological disorders, Phytochemicals

## Abstract

The world population is progressively ageing, assuming an enormous social and health challenge. As the world ages, neurodegenerative diseases are on the rise. Regarding the progressive nature of these diseases, none of the neurodegenerative diseases are curable at date, and the existing treatments can only help relieve the symptoms or slow the progression. Recently, hormesis has increased attention in the treatment of age-related neurodegenerative diseases. The concept of hormesis refers to a biphasic dose-response phenomenon, where low levels of the drug or stress exert protective of beneficial effects and high doses deleterious or toxic effects. Neurohormesis, as the adaptive aspect of hormetic dose responses in neurons, has been shown to slow the onset of neurodegenerative diseases and reduce the damages caused by aging, stroke, and traumatic brain injury. Hormesis was also observed to modulate anxiety, stress, pain, and the severity of seizure. Thus, neurohormesis can be considered as a potentially innovative approach in the treatment of neurodegenerative and other neurologic disorders. Herbal medicinal products and supplements are often considered health resources with many applications. The hormesis phenomenon in medicinal plants is valuable and several studies have shown that hormetic mechanisms of bioactive compounds can prevent or ameliorate the neurodegenerative pathogenesis in animal models of Alzheimer’s and Parkinson’s diseases. Moreover, the hormesis activity of phytochemicals has been evaluated in other neurological disorders such as Autism and Huntington’s disease. In this review, the neurohormetic dose–response concept and the possible underlying neuroprotection mechanisms are discussed. Different neurohormetic phytochemicals used for the better management of neurodegenerative diseases, the rationale for using them, and the key findings of their studies are also reviewed.

## Introduction

Legend has it that in ancient times in the land of Pontus, there was a king who was always afraid to be assassinated. In those days, it was customary to poison kings. This was the least troublesome method of assassination, and usually the person who poisoned the king could not be easily identified. The king found a solution. He ate very small amounts of poison that did not cause him illness or death. The next day he added a very small amount to it. He did so until his body became resistant to the poison. Years later, the king lost a war and decided to commit suicide. But the poison did not cause him death.” According to Paracelsus “all things are poison, and nothing is free of poison; the dosage alone makes it so a thing is not a poison.

The world population is getting older every day. Elderly people require more health care facilities, due to the higher prevalence of chronic diseases, physical disabilities, mental illnesses and other co-morbidities. In this sense, as the world gets older, neurodegenerative diseases are on rise [1]. In most of these neurodegenerative diseases, cell death occurs progressively which usually results in cognitive and functional impairment of the patient. Nowadays, none of the neurodegenerative diseases are curable, and the existing treatments can only help relieve the symptoms or slow the progression of the disease [2]. Hence, the clinicians and researchers should move towards finding new therapies for suffered patients.

Recently, hormesis has increased attention in the treatment of age-related neurodegenerative diseases. The hormesis conception refers to a biphasic dose-response phenomenon, where low levels of the drug or stress exert protective of beneficial effects and high doses deleterious or toxic effects. The concept of “use it or lose it” fits under the umbrella of hormesis when the cells respond adaptively [3]. There are various complex mixtures and specific compounds with potential neuroprotective properties which can exert hormetic responses. In other words, these compounds have toxic effects in high doses but at lower doses they can have a beneficial adaptive effect [4, 5]. The hormetic model shows a more accurate prediction of the dose response compared to the traditional model (for example, linear model without threshold) [6]. These responses typically induce a moderate stimulatory effect that is independent of biological model, cell type, induction factor, and mechanism.

Neurohormesis, defined as the adaptive aspect of hormetic dose responses in neurons, was shown to slow the onset of neurodegenerative diseases and reduce the damage of aging, stroke, and traumatic brain injury. Hormesis was also capable to modulate anxiety, stress, pain, and seizure severity [7]. Universal pharmacological treatments fail in neurodegenerative diseases and this seems to be due to the single mechanism of action of drugs and/or their inability to penetrate neurons [8, 9]. Thus, neurohormesis can be considered as a potentially innovative approach in the treatment of neurodegenerative disorder.

Herbal medicinal products and supplements are often considered health resources with many applications [10, 11]. The hormesis phenomenon in medicinal plants is valuable because it is a guide to understand the underlying mechanism of action. Literally, hormetic models can explain different effects of herbs, including inhibition in higher doses and stimulation in lower doses, which contribute to their regulatory and potential healing aspects of disease [6, 12]. Several studies have been shown that the hormetic effects of bioactive compounds prevent the process of neurodegenerative pathogenesis in animal models such as Parkinson’s and Alzheimer’s diseases [13, 14]. Herein, this paper evaluated the neurohormetic dose–response concept and the possible underlying neuroprotective mechanisms. We also reviewed the available data onon neurohormetic phytochemicals used for a better management of neurodegenerative and other neurologic diseases, the rationale for using them and the key findings of their studies.

### Effects of neurohormetic phytochemicals on immune function

The hormesis theory explains that despite the toxic impacts of high doses of compounds, irradiation, etc., low doses of these elements coul be useful. Furthermore, hormesis helps to remove or at least to reduce the harmful effects of subsequent exposure to higher doses [15]. Phytochemicals hadve a wide diversity of biological properties including antimicrobial, antifungal, antioxidant, and anti-proliferative that permit plants to overcome pests and infectious factors. Phytochemicals are not toxic but also stimulate mild cellular stress responses, when they are consumed by humans at the relatively small doses [16]. Phytochemicals mainly present in fruits and vegetables can decrease the risk of several main disorders including cardiovasculardiseases, cancers, inflammatory and immune diseases and neurodegenerative disorders [17, 18]. At low doses, phytochemical such as flavonoids, catechins, curcumin, resveratrol, quercetin, *ginkgo biloba*, and sulfur compounds present in garlic may assist to improve the immune system [19]. Hormetic phytochemicals including sulforaphane, curcumin, resveratrol, catechins, allicin, and hypericin can stimulate the adaptive stress response signaling pathways enhancing cellular resistance to injury and disease [20].

One of the most studied compounds as an immunomodulator is resveratrol. Recent evidences indicated that resveratrol-induced endpoints showed a hormetic biphasic dose–response relationship [21]. For example, Falchetti et al. [22] investigated the effects of resveratrol on several immune functions of human T-cells in in vitro assay. The findings revealed that in vitro exposure to resveratrol had a biphasic impact on the anti-CD3/anti-CD28-induced development of both IFN-gamma- IL2- and IL4-producing CD8+ and CD4+ T cells, with induction at low resveratrol concentrations and inhibition at high concentrations. Also, resveratrol was foumd to induce a significant increase at low concentrations and decrease at high concentrations of both cytotoxic T lymphocytes (CTL) and natural killer (NK) cells cytotoxic activity. These findings showed the capacity of resveratrol on suppression or upregulation of immune response depending on the concentration [22]. Piceatannol (trans-3,4,3′,5′-tetrahydroxystilbene) extracted from the seeds of *Euphorbia lagascae*, is a structural homolog of resveratrol. It had anti-inflammatory, immunomodulatory and anti-proliferative properties. Piceatannol can inhibit the release of nitric oxide (NO), postaglandin E2 (PGE2) and pro-inflammatory cytokines in a dose-dependent relationship [23]. The immuomodulatory activity of curcumin, the main component of turmeric (*Curcuma longa*), is also well known. It can modulate the activation of T cells, B cells, macrophages, neutrophils, natural killer cells, and dendritic cells [24]. Curcumin can also increase antibody responses at low doses and reduce the expression of proinflammatory cytokines [24, 25]. Because of its capability to modulate the immune system, it had beneficial impacts in different disorders such as arthritis, allergy, asthma, atherosclerosis, Alzheimer’s disease, diabetes, and cancer [24].

### Neurohormesis and aging

Aging is a complex genetic and cellular process with factors are involved, such asoxidative stress, deficiency of protective histones and introns, limited nucleotide excision and recombination DNA repair that accumulated in mtDNA during life [21]. The safety response against stress to maintain survival, adaptation, and stability of health are defined as hemodynamic. The impairment in hemodynamic induces an increase in molecular heterogeneity, alteration of cellular function, and reduction of adaptive stress. The development of an adaptive stress response is associated with an improvement of the hemodynamic structure, the reduction of disease risks, and healthy aging. Hormesis in aging produce biologically beneficial effects and induced protective mechanisms in the cells and the organism [18].

The nuclear erythroid 2-related factor 2 (Nrf2)/ antioxidant response element (ARE) pathway is the one of the most important defensive signaling pathways that controls the expression of antioxidants and phase II detoxifying in response to noxious stimuli The beneficial effects of subtoxic doses of many phytochemicals converge in the Nrf2/ARE pathway, by the activation of upstream pathways including p38, phosphatidylinositol-3-kinase (PI3K), c-jun N-terminal kinase, extracellular signal-regulated protein kinase (ERK), and protein kinase C (PKC) [23].

For example, sulforaphane is an isothiocyanate active ingredient from cruciferous vegetables that is present in high amounts in broccoli seeds and sprouts is the best effective natural compounds in inducing the Nrf2/ARE pathway. Sulforaphane treatment has been reported to activate Nrf2/ARE and induce ARE-driven phase II gene expression such as NAD(P) H Quinone Dehydrogenase 1 (NQO1), inhibit mitogen-activated protein kinases (MAPKs) and NF-κB pathways, activate of ERK1/2 and PI3K/Akt signaling and protect against oxidative stress. The Nrf2/ARE pathway exhibit hormetic dose responses and Nrf2 activation was capable to limit age-related damage [26]. In this sense, curcumin can activate the phase II detoxifying and antioxidant enzymes such as glutathione peroxidase (GPx) hemeoxygenase 1 (HO-1) and glutathione S-transferase (GST) through targeting the Nrf2/ARE pathway and exerting neuroprotective activity. Also, curcumin binds NF- κB, and through this interaction exerts protective effect in the regulation of T-cell-mediated aging [27]. It is also reported that green tea flavonoids such as epigallocatechin gallate, kaempferol, genistein and quercetin can activate the Nrf2 pathway through different mechanisms. Flavonoids can protect against oxidative stress by activating the ERK2/Nrf2/ARE signaling pathway, increasing the levels of HO-1, NQO1, GST, glutamate cysteine ligase catalytic, glutamate cysteine ligase modifier, and modulation of PKC. Quercetin dose-dependently through p38/MAPK and Nrf-2 activation increased the expression of glutathione (GSH), GPx, glutathione reductase (GR), and GST protecting against oxidative stress in aging [28]. EGCG regulates the levels of HO-1 in endothelial cells and increased GST, NQO1 enzymes providing resistance against hydrogenperoxide- induced cell death [29]. Resveratrol treatment caused Nrf2 nuclear translocation and regulation of GST and NQO1 activities in neurons. In age-related disease resveratrol increased intracellular GSH levels and glutamate uptake, and protected against the hydrogen peroxide toxicity. Neuroprotection activity of resveratrol is related to activation of ERK and sirtuin 1 (SIRT1) pathway and reduction the levels of markers of oxidative stress including malondialdehyde (MDA) [30].

The NF-κB and FOXO transcription factors are two pathways that play significant roles in neuronal stress adaptation [31]. The expression of pro-survival genes including Bcl-2 and manganese superoxide dismutase derives from the activation of NF-κB. Resveratrol has been reported to activate FOXO transcription factor which, in turn, regulate genes involved in energy metabolism and antioxidant defense [32].

### Hormesis and neuroprotection mechanisms

Cells are continuously exposed to a harsh environment with a high level of toxic agents. Therefore, the organisms that are able to cope with them will survive successfully [33]. Aging causes several changes to the human brain cells [34]. The pathogenesis of neurodegenerative diseases is characterized by the gradual selective death of vulnerable neuron cells. Although the main factors involved in the process are certain protein aggregation and anatomical vulnerabilities, neurological disorders are associated with underlying processes such as oxidative stress due to the accumulated production of free oxygen radicals throughout the lifespan and which can lead to neuroinflammation and even cell apoptosis [8, 35–37]. Various endogenous and exogenous factors lead to DNA damage, such as reactive oxygen species (ROS) and radiation. Unlike other organs, nervous system is vulnerable to these injuries with limited ability for regeneration. Oxidative DNA damage is believed to be one of the primary detectable events prior to neurodegenerative pathogenesis and may contribute to mitochondrial disorders [38, 39]. Indeed, reduced restorative mechanisms that repairs oxidative damage lesions (base excision repair) may contribute to the development of neurodegeneration. Oxidative DNA attack by ROS can result in the production of more than 20 oxidized base adducts, such as 8-hydroxyguanine (8-OHG), formamidopyrimidines, and 5-hydroxyuracil, being 8-OHG the most widely used marker for analyzing of DNA damage [40].

Hormesis has potential therapeutic effects in repairing DNA damage with low doses of a wide range of factors such as radical scavenging and increase antioxidant activity [41]. Neurohormesis is a new strategy for restricting cellular senescence in which repetitive mild stress exposure such as transcranial electromagnetic treatment (TEMT) shows anti-aging benefits in Alzheimer’s diseases [14]. Moreover, long-term TEMT has the potential against cognitive impairment and neurologic injuries [8].

Another possible pathway that can be considered in the pathogenesis of neurodegenerative diseases is a cytoprotective pathway, in which the expression of genes that encode heat shock proteins (Hsps) as protein chaperones is induced. Hsps can help the cells to resist oxidative stress preventing protein aggregation and facilitating protein folding. However, in addition to their function as protein chaperone, members of the HSP superfamily are involved in processes such as synaptic transmission, autophagy, endoplasmic reticulum stress response, and cell death signaling [36, 37].

**Fig. 1:**
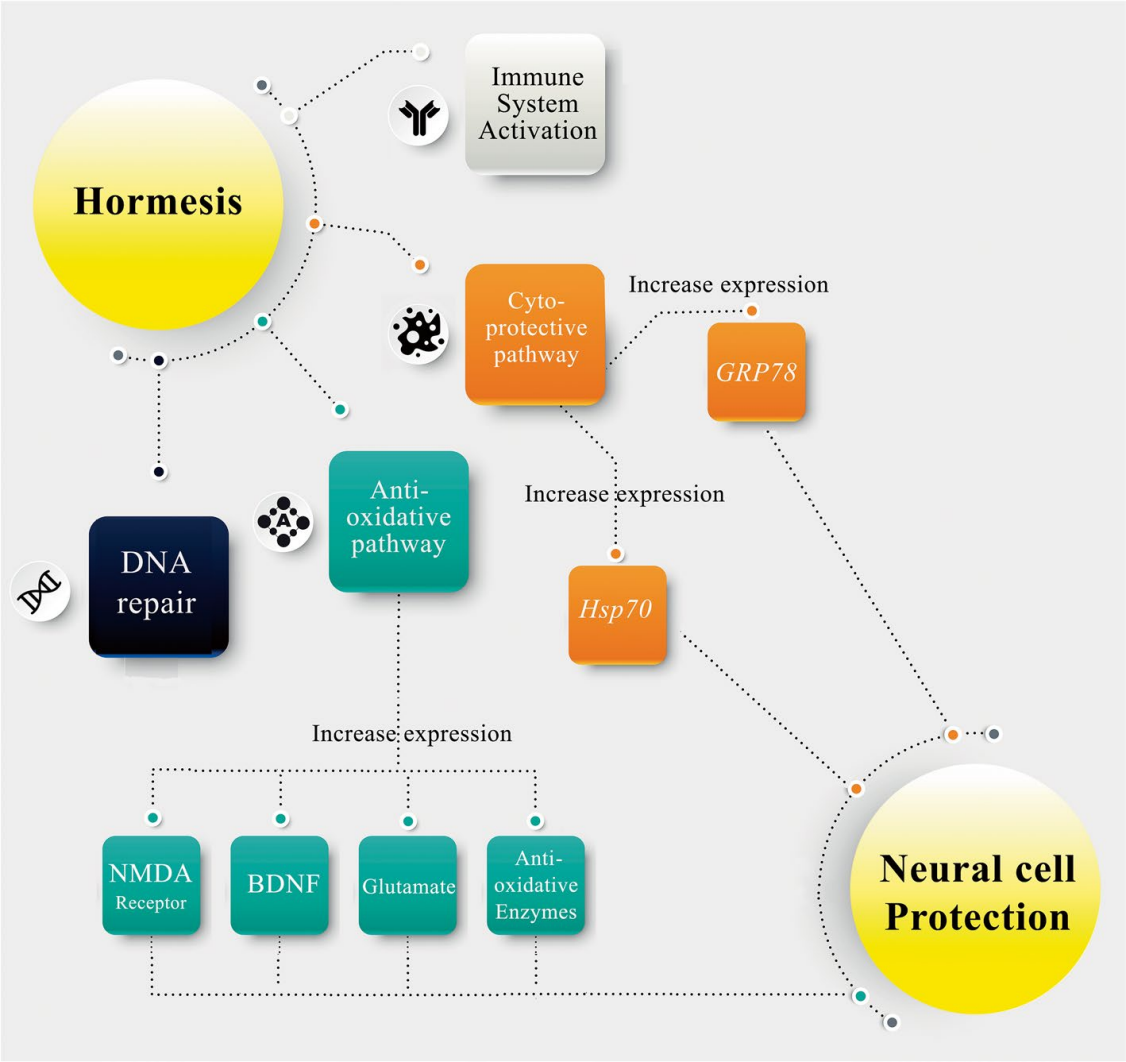
A schematic presentation of the neuroprotection mechanisms of Hormesis. Hsps: heat shock proteins, DNA: deoxyribonucleic acids, GRP: Glucose-Regulated Protein, BDNF: brain derived neurotrophic factor, NMDA: N-methyl-D-aspartate

It should be noted that mild oxidative stresses can increase neurons resistance to more severe stresses by generating mild oxidative stress. This aforementioned mechanism of hormesis includes proteins involved in stabilization of mitochondrial membranes, GSH, activation of brain derived nerve factor (BDNF) and N-Methyl-D-Aspartate (NMDA) receptor, and multiple other anti-oxidative enzymes [5, 42]. A schematic presentation of the neuroprotection mechanisms of hormesis is illustrated in Fig. 1.

### Hormesis and adaptive responses of mitochondria

Mitochondria has a central role in nutrient metabolism, bioenergy production and are essential for cellular homeostasis. Mitochondria-targeting agents in low concentration display protective effects on cellsurvival through ROS-mediated mitohormetic signaling and have a valuable effect on age-related illnesses via mitohormesis [43].

The mitohormesis capability of berberine, an alkaloid isolated from *Coptidis rhizoma* and *Hydrastis Canadensis,* has been examined. A low dose, berberine could target mitochondria via the inhibition of electron transport chain, reduction of energy produced by oxidative phosphorylation and increase in NAD^+^ and ROS. All of the aforementioned pathways increase the adaptability of cells to adverse conditions and caused mitohormesis activity [44]. However, it should be noted that high doses of berberine cause cytotoxicity by affecting DNA synthesis. Other side effects such as diarrhea, emesis, muscular tremor and paralysis, have also been reported with this herbal phytochemical [45]. Asseburg et al. investigated the potential hormetic effects of a polyphenol isolated from grape skin extract (PGE) on age-related dysfunctions of brain mitochondria in C57BL/6 J mice. Administration of PGE at a dose of 200 mg/kg increased brain mitochondrial respiration with a valuable effect on brain adenosine triphosphate (ATP) levels and memory of aged mice through increase in antioxidant activity, signaling pathways involved in energy homeostasis and mitochondrial biogenesis [46]. In C2C12 myoblasts, curcumin ameliorated heat-induced mitochondrial fragmentation through reduction of ROS levels along with increases in NADPH oxidase expression. During heat stress, curcumin at low dose (15 mg/kg) protected the mitochondrial morphology and bioenergetics and, attenuated the heat condition that induced mitochondrial ROS production and tissue injury [47]. In another study, the combination of curcumin (50 and 100 mg/kg) with hesperidin (10 and 25 mg/kg) improved cognition via reduction of caspase-3, MDA and apoptosis and, increase of GSH levels and mitochondrial enzymes [48]. On the other hand, although curcumin seems very safe, high doses have been reported to cause hepatotoxicity, diarrhea, headache, rash, and yellow stool [49]. Therefore, the maximum tolerable dose and half lethal dose of this phytochemical have been disclosed in studies to be 250 and 500 mg/kg, respectively [50].

It has also been reported that resveratrol (10 mg/kg) increased lifespan of mice and exerted mitohormesis activity via enhancement of SIRT1, mitochondrial biogenesis, adenosine monophosphate-activated protein kinase (AMPK), peroxisome proliferator-activated receptor gamma coactivator 1-alpha (PGC-1α) activities and, reduction of insulin-like growth factor 1 (IGF-1) levels [51]. Nevertheless, resveratrol can act as pro-apoptotic and pro-oxidant agent on healthy cells at high doses. Resveratrol has also been linked to cardiac depression and impaired wound healing at these toxic doses [52, 53].

A summary of the hormesis activity of natural compounds in vivo on adaptive responses of mitochondria is provided in Table 1.

**Table 1:** The hormesis activity of natural compounds in vivo in adaptive responses of mitochondria

Activity	Compound	Source Plant	Model	Treatment	Remarks	Reference
Adaptive responses of mitochondria	Berberine	*Coptidis rhizome, Hydrastis canadensis*	Rat	10 μM	Berberine exerts mitohormesis activity	[44]
	Polyphenol	*Ribes nigrum*	Mice	200 mg/kg	Polyphenol increases homeostasis and mitochondrial biogenesis	[46]
	Curcumin	*Curcuma longa*	Rat	15 mg/kg	The low dose was effective	[47]
	Curcumin with hesperidin	*Curcuma longa*	Rat	Curcumin (50, 100 mg/kg), hesperidin (10, 25 mg/kg)	Both compounds improve mitochondrial enzymes and reduce apoptosis	[48]
	Resveratrol	*Ribes nigrum*	Mice	10 mg/kg	Resveratrol exerts mitohormesis activity	[51]

### Hormesis and memory performance

The consumption of epicatechin, a flavanol commonly found in plants, increased the cognition in female C57BL/6 mice. The combination of exercise for 6 weeks and epicatechin (3 mg/kg) improved memory function, hippocampal angiogenesis, and neuronal spine concentration in mice [54]. In another study, epigallocatechin-3-gallate (EGCG) (10 mg/kg), the main polyphenol isolated from green tea, exerted beneficial effects in reversing the cognitive deficit in rats. The underlying mechanism was via modulation of S100B secretion, acetylcholinesterase and antioxidant activity [55]. Various studies have concluded that this phytochemical has a biphasic dose responses. Furthermore, hepatotoxicity and changes in serum lipid profile have been reported at high oral doses [56–58].

The intraventricular injection of resveratrol, a natural component abundant in grapes, improved the long-term memory formation and the long-term potentiation (LTP) induction in 8–9 month-old mice. Resveratrol (2.5 or 5 mg/kg) exerted this beneficial effect via reduction of miR-124 and miR-134 expressions and regulation of cAMP Response Element-Binding Protein (CREB) levels [59]. Shibani et al., investigated the effects of oleuropein, a polyphenol extracted from olive leafs, against memory impairment induced by chronic morphine administration in rats. Oleuropein (15 and 30 mg/kg) treatment improved the spatial learning and ameliorated memory impairments through inhibition of oxidative stress and neuronal apoptosis in the CA1 area of hippocampal neurons of rats [60]. However, it should be noted that this phytochemical, up to 2000 mg/kg, has found to be safe and without serious side effects on the reproductive and developmental organs [61].

A summary of the hormesis activity of natural compounds in vivo on memory performance is provided in Table 2.

**Table 2:** The hormesis activity of natural compounds in vivo in memory performance

Activity	Compound	Source Plant	Model	Treatment	Remarks	Reference
Memory performance	Epicatechin	*Camellia sinensis*	Mice	3 mg/kg	Epicatechin increases cognition	[62]
	Epigallocatechin-3-gallate	*Camellia sinensis*	Rat	10 mg/kg	Epigallocatechin-3-gallate improves cognitive deficit	[55]
	Resveratrol	*Ribes nigrum*	Mice	2.5 or 5 mg/kg	Resveratrol improves the LTP induction	[59]
	Oleuropein	*Olea europaea*	Rat	15 and 30 mg/kg	Oleuropein improves the spatial learning and memory impairments	[60]

### Hormesis and Alzheimer’s disease

Alzheimer’s disease (AD) is the main cause of dementia and its prevalence increases with aging. It is characterized by memory impairment, head injury, neuronal loss and β-amyloid pathology. Amyloid β peptide (Aβ) plays a central role in the neuropathology of AD and oxidative stress may be responsible for the neurotoxicity of Aβ [62, 63].

Cannflavin A, a flavonoid extracted from *Cannabis sativa*, demonstrated hormetic and neuroprotective effects against amyloid β-mediated neurotoxicity in PC12 cells. Cannflavin A, in low concentrations (1 to 10 μM), exerted hormetic effects through inhibition of Aβ neurotoxicity, reduction of Aβ aggregation to PC-12 cells and related neurite loss, while at higher concentrations (> 10–100 μM), exhibited neurotoxicity [64]. Joseph et al. stated that the consumption of blueberries (25 μM) increased memory-associated neuronal signaling, induced modifications in neutral sphingomyelin-specific phospholipase C action and inhibited behavioral deficits in an AD model without any alterations in amyloid beta deposition [65]. Gintonin, extracted from *Panax ginseng,* improved learning and memory dysfunctions in animal models of AD. The orally administration of gintonin (25, 50, or 100 mg/kg) for 3 weeks decreased scopolamine and amyloid-β-induced memory impairment and cholinergic dysfunctions through reduction of acetylcholine concentration, choline acetyltransferase activity and induction of acetylcholine esterase (AChE) activity [66]. It was also reported that ginsenoside Rg1 from *Panax ginseng* exerted protective effects in a mouse animal model of AD. Rg1 (20 mg/kg) ameliorated memory impairment and depression-like behavior via downregulation of complexin-2 (CPLX2), synaptosomal-associated protein 25 (SNP25) and synapsin-2 (SYN2) expression in the hippocampus of mice [67]. Ginseng abuse was reported to cause affective disorders, increase in blood pressure, coagulopathy, bleeding of genital organs, allergy, and liver, kidney, reproductive and cardiovascular system toxicity [68].

The effect of curcumin at low (160 ppm) and high doses (5000 ppm) on oxidative damage, inflammation, and plaque pathology was investigated in mouse model of Alzheimer. Low-dose of curcumin reduced glial fibrillary acidic protein (GFAP), soluble Aβ, insoluble β-amyloid and plaque burden, while higher dose decreased oxidized proteins and interleukin-1β [69]. Furthermore, curcumin encapsulated poly (lactic-co-glycolic acid) (PLGA) nanoparticles, at the dose of 20 mg/kg, improved learning and memory impairments in rat model of Alzheimer. This effect was mediated by stimulation of the Wnt/β-catenin pathway, increase in GSK-3β phosphorylation, modulation of neurogenesis by interaction with Wif-1, Dkk-1, and GSK-3β at very low doses [70]. Resveratrol in a concentration of 25 μM protected the rat hippocampal neurons through inhibition of GF 109203X, activation of protein kinase C (PKC) leading to a reduction of Aβ aggregation [71]. In another study, the protective effect of resveratrol and catechin against β-Amyloid peptide toxicity in PC-12 cells were investigated. The administration of 50 μM catechin and 10 μM resveratrol completely eliminated the toxicity induced by β-Amyloid peptide by interaction with the mitochondrial redox system, modulation of the NF-kB activity and inhibition of calcium ions concentration [72]. In a study conducted by Haque et al., the long-term consumption of green tea catechins ameliorated the cognitive deficits induced by oxidative stress and Aβ. The green tea (5 g/L) exerted beneficial effects against cognitive impairment by reduction of lipid peroxidation in the hippocampus and ROS in the hippocampus and cortex [73]. Sulforaphane, the main compound extracted from cruciferous vegetables, improved cognitive impairment in different doses through reduction in the levels of amyloid-β, tau, inflammation, neurodegeneration and oxidative stress in animal and cell models. The results indicated that the oral administration of sulforaphane, in doses between 10 and 50 mg/kg, displayed the anti-AD-like activity in animal models. In cell models, sulforaphane exhibited anti-AD-like effectiveness at doses of 0.01–10 μM [74]. However, high doses of sulforaphane were reported to cause sedation, hypothermia, disturbance in motor coordination, decreased muscle strength and even death at very high does (200-300 mg/kg) [75]. Urolithin A, a main compound found in pomegranate and walnuts, demonstrated neuroprotective effect in a cellular model of AD. Urolithin A had no effect on autophagy in SH-SY5Y-APP695 cells while exerted hormetic effects through mitochondrial biogenesis which, in turn, induced the transcription of several genes [76]. A summary of the hormesis activity of natural compounds in vitro and in vivo on AD is provided in Table 3.

**Table 3:** The hormesis activity of natural compounds in vitro and in vivo in Alzheimer’s disease

Activity	Compound	Source Plant	Model	Treatment	Remarks	Reference
Alzheimer	Cannflavin A	*Cannabis sativa*	PC12 cells	1 to 10 μM	The low dose was effective	[64]
	Blueberry		Mice	25 μM	The blueberry inhibits behavioral deficits	[65]
	Gintonin	*Panax ginseng*	Mice	25, 50, or 100 mg/kg	Gintonin improves memory dysfunctions	[66]
	Ginsenoside Rg1	*Panax ginseng*	Mice	20 mg/kg	Rg1 ameliorates memory impairment	[67]
	Curcumin	*Curcuma longa*	Mouse	160 ppm	The low dose was effective	[69]
	Curcumin encapsulated PLGA nanoparticles (Cur-PLGA-NPs)	*Curcuma longa*	Rat	20 mg/kg	Cur-PLGA-NPs improve learning and memory impairments	[70]
	Resveratrol	*Ribes nigrum*	Rat	25 μM	Resveratrol reduces Aβ aggregation	[71]
	Resveratrol and catechin	*Ribes nigrum* and *Camellia sinensis*	PC-12 cells	Catechin (50 μM), resveratrol (10 μM)	Catechin is more effective	[72]
	Catechin	*Camellia sinensis*	Rat	5 g/L	Catechin ameliorates cognitive deficits	[73]

### Hormesis and Parkinson’s disease

Parkinson’s disease (PD) is a high prevalent neurodegenerative disorder characterised by loss of dopaminergic neurons in the substantianigra, emotional and olfactory dysfunction and progressive cognitive and motor impairments [77, 78].

Resveratrol has shown to exert neuroprotective effects against 6-hydroxydopamine (6-OHDA)-induced PD in rats. The oral administration of resveratrol (10, 20 and 40 mg/kg) improved chronic inflammation, oxidative stress, mitochondrial dysfunction and reduced the levels of cyclooxygenase-2 (COX-2) and tumor necrosis factor alpha (TNF-α) in the substantianigra [79]. Moreover, resveratrol (100 μM) exerted neuroprotective activity in PD through avoiding cellular oxidative damage reducing dopaminergic neurotoxicity and regulating sirtuin transcription [80]. In another study, the protective effects of resveratrol (20 mg/kg) were examined in a rat model of PD induced by rotenone. The mechanisms responsible for this effect were the regulation of CHOP and, GRP78 genes, the reduction of activated caspase-3, and the induction of GPx and Nrf2 signaling pathways [81].

Levites et al., reported that epigallocatechin 3-gallate (0.1–10 μM) exerted neuroprotective effects in a mice model of PD through inhibition of BCL2 associated X (Bax), MDM2 proto-oncogene (Mdm2), and reduction of B-cell lymphoma 2 (Bcl-2), Bcl-w, and Bcl-xL expression. However, in concentrations higher than 10 μM, epigallocatechin could not counteract the toxic effects of 6-OHDA [82].

The neuroprotective effects of low (10 mg/kg) and high doses (20 mg/kg) of caffeine were evaluated on a rotenone-induced model of PD in rats. Caffeine exerted this effect via improvement of histopathological degeneration and reduction of dopamine concentration [83]. Furthermore, the consumption of caffeine at doses of 60-80 mg/kg has protective properties against rat model of PD by inhibition of nigral dopamine neuron loss, blockage of A_2A_ receptors and reduction of neuroinflammation [84]. Nevertheless, higher caffeine induced psychosis, anxiety, nervousness, and neurobehavioral adverse effect through glutamate excitotoxicity and neuronal death in the brain [85].

In another study, mulberry fruit from *Morus alba* exhibited neuroprotective activity against mouse model of PD via antioxidant and anti-apoptotic properties. The mulberry fruit (500 mg/kg) regulated ROS, NO, Bcl-2 and Bax production and reduced the activation of caspase-3 [86]*.* Toxicity evaluation studies showed ophthalmological abnormality such as conjunctival congestion and also renal tubular pigmentation and discharge coloration at high doses of 4200 mg/kg [9, 87].

In a study conducted by Govindan et al., *Dioscorea alata* tubers improved the health and extend lifespan through hormesis mechanism. The low dose of tubers (100–300 μg/mL) increased the glyoxalase-1, stress protective genes expression and decreased α-synuclein aggregation through SKN-1/Nrf2 and HSF-1 pathways, while higher doses of tubers (400 and 500 μg/mL) exert toxic effects [88, 89].

Ginsenoside Rb1 was used for the management of PD at doses of 10 and 50 μM. It exerted the beneficial effects via inhibition of glutamate excitotoxicity, modulation of synaptic transmission, glutamate receptor expression and improvement of motor functions [90]. In a rat model of PD induced by rotenone, the protective and autophagy modulating activity of quercetin was investigated. Quercetin at a dose of 50 mg/kg significantly improved the behavioral impairment, ER stress, augmented autophagy and reduced Beclin-1 level via decreased oxidative stress [91]. Moreover, oral administration of quercetin (0.3–30 μM) improved the striatal dopamine depletion, behavioral deficits, TH neuronal cell loss and increased mitochondrial biogenesis through activation of the protein kinase D1/Akt cell survival signaling in a mouse model of PD [92]. On the contrary, higher doses of quercetin are associated with cytotoxicity, mutagenicity, alteration in hormone metabolism and also can act as a prooxidant [93, 94].

Brunetti et al., evaluated the protective activity of two main olive oil polyphenols, hydroxytyrosol (250 μg/mL) and oleuropein aglycone (500 μg/mL) using *Caenorhabditis elegans* as a model. Both polyphenols increased locomotion, decreased the accumulation of α-synuclein and prevented the neurodegeneration through hormesis and antioxidative activities. Also, both compounds increased the survival after heat stress, only hydroxytyrosol enhanced the lifespan in unstressed conditions [95]. It should be noted that depending on the dosage, these compounds can be harmful pro-oxidant compounds [96, 97].

Cannabinoids (3 mg/kg) exerted neuroprotective activity against 6-hydroxydopamine toxicity in PD in in vivo and in vitro models. The protective activity of cannabinoids were related to the reduction of TNFα, synthesis of NO, antioxidative and anti-inflammatory properties [98]. It is also reported that allicin (50 μM), an organosulfur agents obtained from garlic, exerted protective effects against 6-hydroxydopamine (6-OHDA)-induced PD. The underlying mechanism for this protective action was related to a reduced LDH release, generation of ROS, lipid peroxidation and leakage of cytochrome c, increased mitochondrial biogenesis, together causing an increase in cell viability, [99].

The neuroprotective activity of curcumin was examined in an animal model of PD. Curcumin, at the dose of 40 mg/kg, exerted this effect through inhibition of α-synuclein accumulation in the dopaminergic neurons, suppression of NF-κB and proinflammatory cytokines, iNOS expression and improvement in the glutathione system [100]. In another study, the neuroprotection of curcumin, naringenin, quercetin and fisetin were evaluated in a model of PD. The results demonstrated that pretreatment with quercetin and fisetin (50 mg/kg) reduced the loss of tyrosine hydroxylase (TH)-positive cells and the loss of dopamine levels. These effects were believed to be related to the antioxidant activities of the compounds [101].

Zhang et al., investigated the hormetic and neuroprotective activity of panaxatriol saponins, extracted from *Panax notoginseng*, in PC12 and zebrafish. Panaxatriol saponins, at low dose, reduced cytotoxicity induced by 6-OHDA and modulated the proliferation of PI3K/Akt/mTOR cell and AMPK/SIRT1/FOXO3 cell survival. These neuroprotective effects may be related to the hormetic effect of the saponins. The low dose of panaxatriol (0.12 mg/mL) could significantly inhibit neuron loss and increase the behavior movement deficiency, whereas high dose (4 mg/mL) displayed neural toxicity [5]. Conversely, high doses of panaxatriol were associated with cardiac toxicity, evidenced as diastolic dysfunction, hypotension and heart failure [5, 102].

Low doses of berberine (0.3, 0.6, 1.3 μM) exerted hormetic and neuroprotective activities through modulation of the PI3K/AKT/Bcl-2 cell survival pathway, the Nrf2/HO-1 antioxidative signaling and improvement of behavior dysfunciot. On the contrary, high dose of berberine (20 μM) did not display neuroprotective effects [103]. Luteolin, a flavonoid present in many plants, displayed hormetic and neuroprotective activity against rotenone-induced toxicity in microglial BV2 cells. The low concentrations of luteolin (1–5 μM) increased cell viability and, the levels of Park2 mRNA, and reduced levels of IL-1β and Lrrk2 mRNA. Luteolin also protected microglia against rotenone compared with a higher dose [104]. In another study, sulforaphane at doses of 1− 10 μM exhibited protective properties in PD. The underlying mechanism responsible for this effect was found to be hormetic and mediated by the activation of Nrf2/ARE and ERK1/2 pathwaya [105]. A summary of the hormesis activity of natural compounds in vitro and in vivo onPD is provided in Table 4.

**Table 4:** The hormesis activity of natural compounds in vitro and in vivo in PD

Activity	Compound	Source Plant	Model	Treatment	Remarks	Reference
Parkinson	Resveratrol	*Ribes nigrum*	Rat	10, 20 and 40 mg/kg	Resveratrol exerts a neuroprotective effect	[79]
	Resveratrol	*Ribes nigrum*	Rat	100 μM	Resveratrol exerts a neuroprotective effect	[80]
	Resveratrol	*Ribes nigrum*	Rat	20 mg/kg	Resveratrol exerts the protective activity	[81]
	Epigallocatechin 3-gallate	*Camellia sinensis*	Mice	0.1–10 μM	Epigallocatechin 3-gallate exerts neuroprotective effects	[82]
	Caffeine	*Camellia sinensis*	Rat	10 mg/kg and 20 mg/kg	The low dose was effective	[83]
	Caffeine	*Camellia sinensis*	Rat	60-80 mg/kg	Caffeine exerts protective activity	[84]
	Mulberry fruit	*Morus alba*	Mouse	500 mg/kg	Mulberry fruit exhibits neuroprotective activity	[86]
	Tubers	*Dioscorea alata*	Mouse	100–300 μg/mL	The low dose was effective.	[88]
	Ginsenoside Rg1	*Panax ginseng*	Mouse	10 and 50 μM	Ginsenoside Rb1 treats of PD	[90]
	Quercetin	*Crocus sativus*	Rat	50 mg/kg	Quercetinexhibits protective activity	[91]
	Quercetin	*Crocus sativus*	Mouse	0.3–30 μM	Quercetin improves behavioral deficits	[92]
	Hydroxytyrosol and oleuropeinaglycone	*Olea europaea*	Mouse	Hydroxytyrosol (250 μg/mL) oleuropeinaglycone (500 μg/mL)	Hydroxytyrosol was more effective	[95]
	Cannabinoids	*Cannabis sativa*	Rat	3 mg/kg	Cannabinoids exerts neuroprotection activity	[98]
	Allicin	*Allium sativum*	PC-12 cells	50 μM	Allicin exerts protective action	[99]
	Curcumin	*Curcuma longa*	Rat	40 mg/kg	Curcumin exhibits protective activity	[100]
	Curcumin, naringenin, quercetin and fisetin	*Curcuma longa, Citrus aurantium, Crocus sativus*	Rat	50 mg/kg	Quercetin and fisetin exhibit neuroprotection effects	[101]
	Panaxatriol saponins	*Panax notoginseng*	PC12 cells	0.12 mg/mL	The low dose was effective.	[5]
	Berberine	*Berberis vulgaris*	PC12 cells	0.3-20 μM	Berberine improves behavior movement deficiency	[103]

### Hormesis and Huntington’s disease

Huntington’s disease (HD) is a genetic neurological illness of the central nervous system that causes clinical manifestations such as progressive choreiformic movements, cognitive impairments, personality disorders, psychiatric deterioration and premature death [106].

The therapeutic potential of protopanaxtriol (Ppt), isolated from *Panax ginseng C.A. Meyer,* was investigated against 3-nitropropionic acid (3-NP)-induced oxidative stress in a rat model of HD. The administration of protopanaxtriol (5,10 and 20 mg/kg) significantly improved behavior, increased the translocation of Nrf2 into the nucleus, reduced the production of free radicals, the expression of NQO1 and HO-1 in striatum [107]. In another study, the administration of Korean red ginseng at doses of 50, 100, and 250 mg/kg/day, exerted therapeutic effects in the inhibition of Huntington’s symptoms. The mechanism of action was related to the inhibition of the phosphorylation of MAPKs and NF-κB pathways. Furthermore, this phytochemical decreased the microglial activation and mRNA expression of TNF-α [108]. Maher et al., have revealed that the oral administration of fisetin and resveratrol (10 μM) was useful for the treatment of HD. Fisetin and resveratrol are very small, available molecules that can cross the blood–brain barrier and activate ERK signaling which, in turn, preserve brain function [109].

In a study conducted by Shivasharan et al., the extracts of *Calendula officinalis* (100 and 200 mg/kg) showed protective activity against 3-NP in rats. The underlying mechanism of this neuroprotective activity was the reduction of oxidative damage, attenuation of behavioral dysfunction, and striatal neuronal loss through its estrogenic, antioxidant and anti-inflammatory activity [110]. Toxicity of this compound at higher doses was manifested with hepatotoxicity [111].

Ramachandran et al., evaluated the effects of thymoquinone (TQ) in comparison with solid lipid nanoparticles of encapsulated thymoquinone (TQ-SLNs) against behavioral alteration, oxidative damage and striatal pathology induced by 3-NP. The study demonstrated that the low dose of TQ-SLNs (10 mg/kg) was highly effective in comparison with the higher dose (80 mg/kg) in the attenuation of oxidative stress, restoration of the antioxidant defense system and protecting the striatal structural microelements [112]. In animal studies, high dose of thymoquinone caused impairments in respiration and hypoactivity [113].

A summary of the hormesis activity of natural compounds in vivo on HD is provided in Table 5.

**Table 5:** The hormesis activity of natural compounds in vivo in HD

Activity	Compound	Source Plant	Model	Treatment	Remarks	Reference
Huntingto	Protopanaxtriol	*Panax ginseng*	Rat	5,10 and 20 mg/kg	Protopanaxtriol improves body weight and behavior	[107]
	Korean red ginseng	*Panax ginseng*	Mice	50, 100, and 250 mg/kg	Korean red ginseng inhibits Huntington’s symptoms	[108]
	Fisetin and resveratrol	*Ribes nigrum*	Mice	10 μM	Both compounds treat of HD	[109]
		*Calendula officinalis*	Rat	100 and 200 mg/kg	*Calendula officinalis* indicates protective effect	[110]
	Thymoquinone and solid lipid nanoparticles encapsulated thymoquinone (TQ-SLNs)	*Nigella sativa*	Rat	TQ (80 mg/kg), TQ-SLNs (10 mg/kg)	The low dose of TQ-SLNs is highly effective	[112]

### Hormesis and autism

Autism spectrum disorders (ASD) are social and communication dysfunctions with no definitive treatment. Observational studies have shown that children with ASD often reveal improvements in behavior and cognition after a febrile condition that is related to altered metabolic pathways. The febrile process caused responses related to cellular stress and induced the expression of Hsps. Several agents including sulforaphane and hydroxytyrosol were reported to induce metabolic impacts in cellular stress responses similar to fever [114]. Sulforaphane can upregulate Hsps and other mechanisms such as synaptic transmission that may lead to cortical connection improvement [115]. These functions have been established to be decreased in ASD [116]. Furthermore, the compound has protective impacts against several neurodegenerative disorders via induction of the Nrf2 and heat shock factor 1 (HSF1)-dependent genes. On the other hand, it is an effective in maintaining proteome in stressful situations and activator of Hsp that act similar to fever. Several findings indicated that febrile illness may reduce special behavioral and cognitive areas in ASD patients [115, 117]. Furthermore, fever can upregulate Hsps that may improve depressed cortical connectivity in ASD [116]. Overall, sulforaphane is recognized as a minimally toxic agent with ability to improve abnormalities with ASD, including oxidative stress, oxidative phosphorylation, GSH synthesis, mitochondrial action, and neuroinflammmation [117]. The discovery of the hormesis phenomenon appears to make insights in the development of new approaches in vitro*/*in vivo and clinical aspects of prevention and therapy of ASD and other neurologic diseases. The hormesis activity of phytochemicals, including sulforaphane and hydroxytyrosol are lacking, althoguh several clinical studies have evaluated the effect of sulforaphane in patients with ASD.

In a randomized controlled trial (RCT), 44 pediatric children with moderate to severe ASD received 50–150 μmol oral daily doses of sulforaphane from broccoli sprout extracts for 18 weeks, followed by four weeks without treatment. Three valid behavioral questionnaires including the Social Responsiveness Scale (SRS), Aberrant Behavior Checklist (ABC), and Clinical Global Impression Improvement Scale (CGI-I) were used. This study revealed statistically significant improvements in behavior in the intervention group in comparison to the control (34% decrease in ABC and 17% decrease in SRS scores). Moreover, social interaction, abnormal behavior, and verbal communication were improved (*p* = 0.015 and *p* = 0.007). However, after sulforaphane discontinuation, these scores returned to baseline levels [117]. In another RCT, 60 children with ASD were randomized to receive risperidone plus sulforaphane or placebo. The patients received sulforaphane at daily dose of 50 μmol (≤45 kg) or 100 μmol (> 45 kg). The patients were evaluated by ABC-community edition at first and after 5 and 10 weeks. Sulforaphane group revealed higher improvements in irritability score (*p* = 0.001) and hyperactivity/noncompliance score (*p* = 0.015). Also, there was statistically significant time and treatment effects for irritability and hyperactivity/noncompliance. Other secondary measures were not different between groups. This study showed the effect of addition of sulforaphane to risperidone for the management of irritability and hyperactivity symptoms in these patients [118]. Another phase-2 RCT assessed the effect of sulforaphane in 50 children with ASD. Ohio Autism Clinical Global Impressions Scale-Severity and Improvement (OACIS-S and I), SRS and ABC were completed at each visit. In preliminary analysis, the OACIS-I score improved in 26% of patients at week 7, 38% at week 15, 64% at week 22, and 64% at week 30. The most frequent side effects of sulforaphane include the following: insomnia (17%), flatulence (15%) and constipation (13%) [119]. A 28-week follow-up study including only six patients was performed by Evans and Fuller (2016).. In this study, each patient received sulforaphane extracted from broccoli sprout and were evaluated by certain attributes associated with ASD symptoms. Among 92 attributes which were determined as moderate to severe or severely influenced by their ASD, 80% showed positive effects and 39% showed significant improvement. No adverse events were reported in this study [120].

An open-label study was conducted to explore the urinary metabolites of sulforaphane which were correlated with clinical improvements in 15 ASD children. ABC and SRS scores and fasting urinary metabolites were assessed pre and post intervention. After 12 weeks, SRS score showed a significant change from baseline, while ABC score improved during the study course. They also determined 77 urinary metabolites which presented correlation with symptom changes and were categorized in oxidative stress, gut microbiome or neurotransmitters, hormones, amino acid, and sphingomyelin metabolism pathways [121]. In one animal study, the therapeutic effects and molecular mechanisms of sulforaphane were evaluated in asocial BTBR mice and its social counterpart C57/BL6 (C57) mice. This study showed that BTBR receiving sulforaphane had lower self-grooming/marble burying behavior, and higher social interaction in comparison to untreated BTBR mice. Furthermore, sulforaphane caused a reduction in Th17 immune responses (STAT3, RORC, IL-17 A and IL-23R expression in CD4+ T cells), and oxidative stress variables in neutrophils/cerebellum (NFkB, iNOS, and lipid peroxides). It seems that sulforaphane by activation of Nrf2 can alter the dysfunction of Th17 immune cells and the imbalance between oxidant-antioxidant mechanisms in BTBR mice [122]. Another RCT was performed to assess the effect of sulforaphane in 57 children with ASD during 36 weeks. This trial was carried out in three phases: in phase 1 (1 to 15 weeks), the patients were assigned to receive either sulforaphane or placebo. In phase 2 (16 to 30 weeks), all of the patients received sulforaphane and, in phase 3 (31 to 36 weeks), there was no intervention. The findings showed that the total score of Ohio OACIS-S and I were not significantly different between the two arms at 7 and 15 weeks. At 15 weeks, ABC score, based on the rating of caregiver, improved significantly, with no changes in SRS-2. GSH redox biomarkers, mitochondrial respiration, inflammatory parameters and Hsps had a significant change in sulforaphane versus placebo [123]. In another study, molecular markers were evaluated in peripheral blood in mononuclear cells of healthy donors and autism patients in response to sulforaphane. The results showed an increase in mRNA levels of cytoprotective enzymes and Hsps. In addition, the mRNA levels of pro-inflammatory markers including IL-1β, IL-6, COX-2 and TNF-α were decreased [124]. At the end of this study, among 16 patients, one experienced a lasting behavioral improvement even after stopping sulforaphane, and another ten improved during sulforaphane treatment [125]. A summary of the hormesis activity of natural compounds in vivo on autism is provided in Table 6.

**Table 6:** The hormesis activity of natural compounds in vivo in autism

Activity	Compound	Source Plant	Model	Treatment	Remarks	Reference
Autism	Sulforaphane	*–*	BTBR and C57 mice	50 mg/kg, i.p. once daily for seven days	Sulforaphane improved autism-like symptoms in BTBR mice	[122]

### Hormesis and drug reaction of neurological medications

Many drugs display a hormetic biphasic dose–response relationship. In other words, their beneficial effects increase to some low dose levels, while decrease at higher doses. Anxiolytic, anti-seizure, anti-tumor drugs are well-known examples of drugs with hormetic properties. For instance, in AD, physostigmine (a natural component of the Calabar bean, *Physostigma venenosum*) was administered over a wide range of doses into the bright and dull mice for prevention of the normal hydrolysis of the acetylcholine. In this study, both the dull and bright mice revealed the U-shaped feature of dose–response association. Thereafter, several drugs have been extensively evaluated for this anti-acetylcholinesterase concept, including tacrine, heptylphosphostigmine, huperizine A, arecoline and gastigmine. These drugs have also shown an U-shape dose-response relationship in several animal models [126]. Moreover, various endogenous agonists including neurosteroids, peptides such as cholecystokinin octapeptide (CCK-8), vasopressin, neuropeptide Y, and miscellaneous agents (e.g., platelet activity factor, epinephrine, and nicotinic receptors antagonists), also presented an hormetic biphasic dose-response relationship for memory [127]. Notably, four out of the five drugs approved by US FDA forthe treatment of AD, donepezil, galantamine, rivastigmine, tacrine, revealed the hormetic dose–response. Memantine, an N-methyl-D-aspartate (NMDA) antagonist, the latest approved drug, also acts through an hormetic-like inverted U-shaped dose response association [126]. With regard to concerns about long-term adverse effects with levodopa, including end-of-dose or tachyphylactic worsening of symptoms and signs, and dyskinesia, new treatment strategies for PD are being developed [128]. These adverse effects lead to the introduction of other pharmacologic approaches including low dose of radiation, and herbal extracts. The literature has determined nearly 50 agents with the power to prevent some adverse effects of PD in one or more experimental models. Most of these agents are of herbal origin as an endogenous (e.g., estrogen, creatine, orexin, oleoylethanolamide) or synthetic source (e.g., lactacystin, apomorphine, and glucose oxidase). Regardless of the underlying mechanisms of PD treatments (such as increases in GSH or ATP), maximal protection rate, and response patterns revealed similarities to the hormetic biphasic dose-response feature. On the other hand, most of the compounds that protect against PD damage have also been assessed for their ability to influence other neurodegenerative disorders (such as AD and HD), often with similar success by inducing antioxidant responses. These results showed that several agents with the potential to prevent or reduce PD-like effects have hormetic responses. These agents have stimulatory impact at range of 30-60% and inhibition impact at higher doses. The effective dose also has a wide range of variability [9]. In this sense, levodopa that can alleviate motor symptoms, has reported to show an hormetic response [129]. This is because low cumulative dose led to sustained clinical efficacy several days after levodopa treatment, while at higher doses it has toxic effects [130]. It was also demonstrated that levodopa can influence multiple cognitive functions in a process mediated by mesocortical dopaminergic pathways [131]. Actually, levodopa had hormetic-like U-shaped dose-response. Furthermore, recent studies revealed that neuroinflammatory processes have an essential role in the progression of PD. In these events, the activation of proinflammatory microglial M1 phenotype may be induced by cytokines. Several studies showed that the progression of PD could be attenuated by neuroprotective agents (such as donepezil or rosiglitazone) with hormetic effects keeping the anti-inflammatory M2 phenotype in microglia [132, 133].

## Conclusions and future prospects

Neurohormesis, the adaptive aspect of the hormetic dose-response in neurons, appears to have potential benefits in neurodegenerative and other neurological disorders. The hormesis phenomenon attributed to several medicinal plants is valuable, since hormetic models can explain different aspects of these herbs and how they exert inhibitory effects at high doses and stimulation at low doses to ameliorate or cure a wide range of disorders. The low dose of plant extracts or plant-derived compounds exerts a significant effect in aging-relative illnesses through mitohormesis, while high doses increase the respiration rate of brain mitochondria.

Supplemental administration of phytochemicals helps improve spatial learning and memory impairments at lower doses. While the low doses of these chemicals exert hormetic effects in AD and PD, higher concentrations were associated with neurotoxicity. Both low and high doses of phytochemicals have shown therapeutic effects in HD with low doses being more effective compared to higher doses. The hormesis activity of phytochemicals in ASD is lackingand further clinical studies are required in this field.

In the present review, we attempted to bring up all the existing literature, both in vitro and in vivo studies, on neurohormetic phytochemicals for the better management of neurodegenerative diseases and other neurologic disorders, the rationale for using them and the key findings of the studies. We aimed to attract the attention of the world’s scientists to the important role of neurohormetic properties of phytochemicals in the management of neurodegenerative and other neurologic disorders. Because of significant inter-individual diversity in response to pharmacological agents, further clinical studies are demanded on the effectiveness of phytochemicals in other neurological disorders, such as multiple sclerosis, amyotrophic lateral sclerosis. Future clinical and animal studies are crucial to find the specific mechanism for mitohormesis activities of natural products.

## Data Availability

Not applicable.
